# Neuropsychiatric Tolerability of Long-Acting Cabotegravir and Rilpivirine in People With HIV With Prior INSTI-Related CNS Toxicity: A Real-World Cohort Study

**DOI:** 10.1093/ofid/ofag210

**Published:** 2026-04-09

**Authors:** Marta Rosas Cancio-Suárez, Ana Moreno, Santos Del Campo Terrón, María Jesús Vivancos, Manuel Vélez Díaz-Pallarés, José Luis Casado, Carmen Cano, Roser Navarro-Soler, Santiago Moreno, María Jesús Pérez-Elías

**Affiliations:** Department of Infectious Diseases and IRYCIS, Hospital Universitario Ramón y Cajal, Madrid, Spain; CIBERINFEC, Instituto de Salud Carlos III, Madrid, Spain; Department of Medicine, University of Alcalá de Henares, Alcalá de Henares, Spain; Department of Infectious Diseases and IRYCIS, Hospital Universitario Ramón y Cajal, Madrid, Spain; CIBERINFEC, Instituto de Salud Carlos III, Madrid, Spain; Department of Infectious Diseases and IRYCIS, Hospital Universitario Ramón y Cajal, Madrid, Spain; Department of Infectious Diseases and IRYCIS, Hospital Universitario Ramón y Cajal, Madrid, Spain; CIBERINFEC, Instituto de Salud Carlos III, Madrid, Spain; Pharmacy Department, Hospital Universitario Ramón y Cajal, Madrid, Spain; Department of Infectious Diseases and IRYCIS, Hospital Universitario Ramón y Cajal, Madrid, Spain; CIBERINFEC, Instituto de Salud Carlos III, Madrid, Spain; Department of Infectious Diseases and IRYCIS, Hospital Universitario Ramón y Cajal, Madrid, Spain; CIBERINFEC, Instituto de Salud Carlos III, Madrid, Spain; Department of Infectious Diseases and IRYCIS, Hospital Universitario Ramón y Cajal, Madrid, Spain; CIBERINFEC, Instituto de Salud Carlos III, Madrid, Spain; Department of Infectious Diseases and IRYCIS, Hospital Universitario Ramón y Cajal, Madrid, Spain; CIBERINFEC, Instituto de Salud Carlos III, Madrid, Spain; Department of Medicine, University of Alcalá de Henares, Alcalá de Henares, Spain; Department of Infectious Diseases and IRYCIS, Hospital Universitario Ramón y Cajal, Madrid, Spain; CIBERINFEC, Instituto de Salud Carlos III, Madrid, Spain

**Keywords:** cabotegravir, integrase inhibitors, long-acting ART, neuropsychiatric adverse events, rilpivirine

## Abstract

**Background:**

Neuropsychiatric adverse events (NPAEs) are a recognized limitation of oral integrase inhibitors (INSTIs). Long-acting cabotegravir/rilpivirine (CAB/RPV LA) has expanded treatment options for people with HIV (PWH), but evidence regarding its neuropsychiatric tolerability in those with prior INSTI-related CNS toxicity remains limited.

**Methods:**

We conducted a retrospective–prospective cohort study at a tertiary HIV center in Madrid, including all PWH initiating CAB/RPV LA between January 2023 and March 2025. Participants were stratified by documented history of neuropsychiatric intolerance to oral INSTIs. The primary outcome was the incidence of new or recurrent NPAEs following CAB/RPV LA initiation; the secondary outcome was treatment discontinuation due to neuropsychiatric intolerance.

**Results:**

Of 393 patients initiating CAB/RPV LA, 36 (9.2%) had discontinued a prior oral INSTI due to CNS toxicity. New or recurrent NPAEs occurred in 12 individuals (3.1%), corresponding to an incidence density of 2.2 events per 100 person-years (95% CI, 1.1–3.6). Patients with prior INSTI-related neurotoxicity had a significantly higher risk of NPAEs after switching to CAB/RPV LA (19% vs 1.4%; *P* < .001) and a greater rate of discontinuation due to neuropsychiatric intolerance (14% vs .8%; *P* < .001). All discontinuations led to complete symptom resolution within 6–8 weeks of CAB/RPV LA withdrawal.

**Conclusions:**

A history of CNS intolerance to oral INSTIs was associated with recurrence of neuropsychiatric symptoms and treatment discontinuation under CAB/RPV LA. These findings underscore the importance of individualized risk assessment and proactive neuropsychiatric monitoring when prescribing long-acting injectable ART to susceptible patients.

Lifelong adherence to daily oral antiretroviral therapy (ART) remains a challenge for many people with HIV-1 (PWH), with pill fatigue, stigma, and unintentional disclosure contributing to missed doses and virologic failure. Long-acting (LA) injectable ART—currently formulated as intramuscular cabotegravir plus rilpivirine (CAB/RPV) administered every 8 weeks—offers a transformative alternative that improves convenience, supports adherence, and reduces treatment-related stigma [1, 2]. Pivotal phase 3 trials (ATLAS, FLAIR, and ATLAS-2M) and early real-world cohorts consistently show that CAB/RPV LA is non-inferior to oral ART for maintaining virologic suppression; the most common adverse events are mild, transient injection-site reactions, while neuropsychiatric adverse events (NPAEs) such as headache or fatigue are uncommon and rarely lead to treatment discontinuation [3–5]. A recent systematic review confirmed that the neuropsychiatric safety profile of CAB/RPV LA is broadly comparable to conventional oral regimens [4].

Despite belonging to the integrase strand transfer inhibitor (INSTI) class, the neuropsychiatric safety profile of cabotegravir/rilpivirine (CAB/RPV LA) remains poorly characterized in patients with prior INSTI-related CNS intolerance. While observational data suggest increased reports of CNS symptoms with certain INSTIs (notably dolutegravir) and lower incidence in others (such as bictegravir), the evidence is heterogeneous and does not support a definitive ranking of tolerability across the class. Importantly, intolerance to 1 INSTI does not invariably predict intolerance to another, indicating that neurotoxicity may be molecule-specific rather than a pure class effect [6–10].

The administration of an LA injectable raises unique concerns in patients with prior INSTI-related central nervous system (CNS) toxicity. Once injected, CAB persists for weeks; thus, any adverse CNS effects are not readily reversible. Current guidance therefore advocates careful neuropsychiatric assessment before initiating LA therapy and vigilant post-injection monitoring, particularly in individuals with a history of NPAEs on oral INSTIs [6, 8, 11]. To date, however, data on the neuropsychiatric tolerability of CAB/RPV LA in this high-risk subgroup are limited. Whether prior CNS intolerance predicts recurrence with LA formulations is a pressing clinical question with direct implications for patient selection, counseling, and safety monitoring.

We conducted a retrospective–prospective cohort study at a tertiary HIV center in Madrid to characterize the neuropsychiatric safety of CAB/RPV LA in routine care, with a specific focus on PWH who had previously discontinued an oral INSTI because of NPAEs. Our study aims to inform evidence-based decision-making for the expanding use of LA injectable ART.

## METHODS

We conducted an observational cohort study at the Infectious Diseases Unit of Hospital Ramón y Cajal, Madrid, a tertiary referral center with a large cohort of people living with HIV. The study included both retrospective and prospective data collection.

All patients who initiated long-acting CAB/RPV between January 2023 and March 2025 were eligible. All patients were aged over 18 years and started CAB/RPV under clinical criteria.

The analysis compared patients who developed NPAEs after CAB/RPV LA initiation with those who did not. NPAEs were defined as the occurrence of new or recurrent CNS or psychiatric symptoms temporally associated with CAB/RPV LA initiation, including but not limited to insomnia, anxiety, depressive symptoms, mood changes, irritability, agitation, headache, dizziness, fatigue, sleep disturbances, or cognitive complaints. NPAEs were identified during routine clinical visits through clinician documentation and patient self-report. Symptoms could be either spontaneously reported or actively elicited as part of standard follow-up assessments.

Prior CNS intolerance to oral INSTIs was examined as a predictor of NPAE occurrence. The primary outcome was the incidence rate of new or recurrent NPAEs following initiation of CAB/RPV LA. The secondary outcome was discontinuation of CAB/RPV LA due to neuropsychiatric intolerance.

Data were collected by reviewing medical records, clinician reports, and patient interviews during routine follow-up. The extracted variables included demographics (age, sex at birth, and country of origin); HIV-related parameters: (diagnosis date, CD4+/CD8 + counts, nadir CD4+, viral load, ART history, and prior INSTI exposure); CAB/RPV LA treatment details (initiation date, dosing schedule, and number of injections); neuropsychiatric history (type, severity, timing of prior NPAEs, psychiatric comorbidities, and psychotropic medications); and outcomes (new neuropsychiatric symptoms post-initiation, treatment discontinuation and reason, referral to mental health services).

Information on substance use (including alcohol, cannabis, and other recreational drugs) was reviewed at the time of switching to long-acting cabotegravir/rilpivirine. None of the patients had active substance use at the time of the switch. Substance use was therefore not included in the primary analysis.

Virologic suppression was defined as HIV-1 RNA <50 copies/mL and was assessed before CAB/RPV LA initiation and throughout follow-up. Adherence to follow-up was assessed based on attendance to scheduled injection visits. Delayed injections were defined as administration occurring more than 7 days after the scheduled date.

Use of an oral lead-in prior to CAB/RPV LA initiation was not performed in this cohort, consistent with local clinical practice.

### Statistical Analysis

Categorical variables were summarized using frequencies and percentages; continuous variables were expressed as medians with interquartile ranges or means with standard deviations, as appropriate. Group comparisons were made using the χ^2^ test or Fisher's exact test for categorical variables and the Student's *t*-test or Mann–Whitney *U* test for continuous variables, depending on distribution.

Multivariable logistic regression models were constructed to estimate adjusted odds ratios for the association between prior INSTI-related neuropsychiatric toxicity and incident NPAEs, adjusting for age, sex, prior psychiatric history, and comorbidity burden. Incidence rates were calculated per 100 person-years (PY) with exact Poisson CIs.

All statistical analyses were performed using Stata version 18.0 (StataCorp LLC). A two-sided *P*-value <.05 was considered to indicate statistical significance.

### Ethical Considerations

The study was conducted following the principles of the Declaration of Helsinki and approved by the Institutional Review Board (Comité de Ética de la Investigación con Medicamentos) of Hospital Universitario Ramón y Cajal.

## RESULTS

Between January 2023 and March 2025, a total of 393 PWH initiated long-acting cabotegravir/rilpivirine (CAB/RPV LA) at our center. The cohort consisted predominantly of virologically suppressed individuals with stable ART before switching.

Among them, 36 patients (9.2%) had a documented history of neuropsychiatric toxicity attributed to prior oral integrase inhibitor (INSTI) therapy that had led to treatment discontinuation. These individuals were compared with the remaining 357 patients who had no known history of CNS intolerance to INSTIs.

Baseline characteristics are summarized in Tables 1 and 2. Compared with patients without prior CNS toxicity, those with a history of NPAEs were more likely to be women (25% vs 11%, *P* = .028), older (mean age 51 ± 12 vs 44 ± 12 years, *P* = .001), and had a longer duration of ART exposure (mean 15 ± 7 vs 11 ± 8 years, *P* = .003). They also had more ART regimen switches (mean 7 ± 4 vs 4 ± 4, *P* = .001), a higher burden of comorbidities (83% vs 51%, *P* = .001), and were less likely to have been on an INSTI-based regimen immediately prior to switching to CAB/RPV LA (50% vs 85%, *P* < .001). CD4+ cell counts and history of AIDS-defining illnesses were comparable between groups.

**Table 1.** Baseline Characteristics of the Total Cohort Initiating CAB/RPV LA (*n* = 393)

Characteristic	Value
Age, median (range), y	43.0 (22–76)
≥50 y, *n* (%)	135 (34%)
Female sex, *n* (%)	48 (12%)
HIV risk factor, *n* (%)	MSM 304 (77%)
Preswitch ART regimen, *n* (%)	INSTI: 323 (82%) NNRTI: 59 (15%) PI: 11 (3%)
Duration of ART preswitch, median (range), y	9 (0.5–35)
Number of prior ART regimens, median (range)	3 (1–21)
≥5 ART regimens, *n* (%)	119 (30%)
AIDS diagnosis, *n* (%)	61 (15%)
CD4+ cell count, median (range), cells/μL	710 (31–2089)
Undetectable HIV viral load, *n* (%)	385 (98%)
BMI, median (range), kg/m^2^	25 (16–43)
BMI ≥ 30, n (%)	44 (11%)
HIV subtype (available in 152 patients), *n* (%)	Subtype B: 130 (85%) Subtype A: <2%
Comorbidities, *n* (%)	212 (54%)
– Hypertension	46 (12%)
– Dyslipidemia	49 (12%)
– Psychiatric follow-up	37 (9%)
– Cancer	13 (3%)
Concomitant therapies^a^, *n* (%)	194 (49%)
Initiation of CAB/RPV LA proposed by, *n* (%)	Physician: 50% Patient: 46%
Previous NNRTI use, *n* (%)	223 (60%)
NNRTI resistance mutation, *n* (%)	8 (2%)
Documented mutations	Y181C (*n* = 1), K103N (*n* = 5), N348 + V108I (*n* = 1), V106I (*n* = 1)
HBsAb positive, *n* (%)	313 (80%)
Isolated HBcAb, *n* (%)	20 (5.1%)

Note: ^a^Concomitant therapies refer to nonantiretroviral chronic medications.

Abbreviations: MSM, Men who have sex with men; ART, antiretroviral therapy; INSTI, integrase strand transfer inhibitor; NNRTI, non-nucleoside reverse transcriptase inhibitor; PI, protease inhibitor; BMI, body mass index; HBsAb, hepatitis B surface antibody; HBcAb, hepatitis B core antibody.

**Table 2.** Baseline Characteristics of Patients With and Without Prior CNS Toxicity Attributed to INSTIs

	CNS Toxicity By Previous INSTI (*n* = 36)	No CNS Toxicity By Previous INSTI (*n* = 357)	*P*
Women (*N*, %)	9 (25%)	39 (11%)	.028
Age (mean ± SD)	51 ± 12	44 ± 12	.001*
BMI (mean ± SD)	25 ± 4	26 ± 4	NS
Time on ART (y, mean ± SD)	15 ± 7	11 ± 8	.003*
Mean *N* of prior ART lines (*n*, % ± SD)	7 ± 4	4 ± 4	.001*
AIDSSIDA (%)	19	15	NS
CD4 No. (cells/µL, mean ± SD)	750 ± 229	757 ± 326	NS
Comorbidities (%)	83	51	.001*
Prior INSTI ART before switch to C/R (%)	50%	85%	.0001*
CNS toxicity with C/R (*N*, %)	7 (19)	5 (1,4)	.0001*
Suspension C/R for CNS toxicity (*N*, %)	5 (14)	3 (0,8)	.0001*

Note: *p < 0.05.

During a cumulative follow-up of ∼536 PY, 12 patients (3.1%) developed new or recurrent NPAEs, corresponding to an incidence of 2.2 per 100 PY (95% CI, 1.1–3.6). Incidence was markedly higher in those with prior INSTI-related toxicity (19% vs 1.4%; *P* < .001), who also had higher rates of treatment discontinuation for neuropsychiatric intolerance (14% vs 0.8%; *P* < .001). Among the 8 individuals discontinuing CAB/RPV LA, symptoms included insomnia, anxiety, and mood disturbances. As shown in Table 3, 5 of these individuals were reinitiated on their prior oral INSTI-based regimen, while 3 were transitioned to non-INSTI-based regimens (2 with doravirine, 1 with a boosted protease inhibitor). In all cases, neuropsychiatric symptoms resolved completely within 6–8 weeks of discontinuation.

**Table 3.** Clinical Characteristics of Patients Who Discontinued CAB/RPV LA Due to Neuropsychiatric Adverse Events (*n* = 8)

Patient ID	Sex	Age (y)	Prior INSTI Tolerance	Neuropsychiatric Symptoms On CAB/RPV LA	Time To Onset (wks)	Subsequent Regimen	Symptom Resolution
P01	Male	35	No	Insomnia, anxiety	4	Doravirine-based	Yes (6 wks)
P02	Female	63	Yes, BIC	Mood swings, insomnia	8	PI-based regimen	Yes (8 wks)
P03	Male	61	Yes, DTG	Irritability, restlessness	6	Doravirine-based	Yes (6 wks)
P04	Male	72	Yes, DTG and BIC	Headache, dizziness	4	PI-based regimen	Yes (6 wks)
P05	Female	70	No	Anxiety, depressed mood	4	DTG-based	Yes (8 wks)
P06	Male	63	No	Insomnia, agitation	3	BIC-based	Yes (6 wks)
P07	Female	56	Yes, BIC	Sleep disturbance, anxiety	3	PI-based regimen	Yes (7 wks)
P08	Male	52	Yes, BIC	Fatigue, low mood	2	PI-based regimen	Yes (8 wks)

No patient required psychotropic medication beyond standard supportive care, and all maintained full virologic suppression on their new oral regimens.

Viral suppression was maintained in >98% of patients before switching and throughout follow-up, including among those who discontinued CAB/RPV LA due to NPAEs.

## DISCUSSION

In this real-world cohort, we found that PWH who had previously discontinued oral INSTIs due to CNS toxicity exhibited a significantly higher incidence of NPAEs after initiating CAB/RPV LA, as well as an increased risk of discontinuation for this reason. These findings suggest that prior INSTI-related neurotoxicity is not an isolated or irrelevant event but may reflect a persistent individual susceptibility to CNS effects that can recur, even with different formulations or pharmacokinetic profiles.

Our results are consistent with recent pharmacovigilance alerts and observational data indicating that depressive symptoms, insomnia, anxiety, and mood alterations—although generally uncommon—can occur under CAB/RPV LA, particularly in predisposed individuals [12, 13]. The U.S. Food and Drug Administration has highlighted that depressive disorders, including suicidal ideation and suicide attempts, have been reported in post-marketing surveillance of cabotegravir and rilpivirine, leading to warnings about the need for prompt clinical assessment and ongoing monitoring in at-risk populations [14]. Similarly, current guidelines from the Infectious Diseases Society of America and the International Antiviral Society–USA (IAS–USA) recommend routine screening for depression and anxiety in all PWH, and more frequent assessment when initiating long-acting ART, particularly in those with psychiatric history or prior antiretroviral-related NPAEs [15].

Our data reinforce these recommendations. Among individuals with prior oral INSTI intolerance, the recurrence of CNS symptoms under CAB/RPV LA—though still limited in absolute terms—was significantly more frequent than in those without such a history. Moreover, this subgroup showed a disproportionately higher rate of treatment interruption due to neuropsychiatric events. These findings argue against a purely molecule-specific explanation and support the notion that CNS vulnerability may be a patient-related trait influenced by underlying neurobiology, psychiatric comorbidity, or pharmacogenetic variation [16–18].

Although most neuropsychiatric symptoms were mild or moderate, their clinical relevance lies in their impact on treatment continuity and patient quality of life. Long-acting injectable regimens, by virtue of their extended half-life and irreversibility once administered, require a proactive approach to neuropsychiatric monitoring. In our view, it would be useful for clinicians to perform mental health screening (eg, PHQ-9 and GAD-2) prior to initiation and reassess regularly at each injection visit. Patients with known psychiatric illness, previous neuropsychiatric ART intolerance, or other psychosocial vulnerabilities may benefit from closer follow-up and early referral to mental health services if symptoms emerge.

This study has limitations. It was conducted at a single center with a modest sample size, which may limit generalizability and preclude the detection of very rare events. Symptom reporting was based on clinician documentation, and structured patient-reported outcome measures were not systematically used. Substance use was not consistently documented, and residual confounding cannot be excluded. Individual-level time-to-event data were not systematically available, precluding formal survival analyses such as Kaplan–Meier estimates. However, the strength of our work lies in its focus on a high-risk group often excluded from pivotal trials, and in its ability to quantify both symptom burden and treatment discontinuation attributable to CNS intolerance.

Our findings suggest that a history of CNS-related intolerance to oral INSTIs is associated with a higher risk of recurrent neuropsychiatric symptoms and treatment discontinuation following initiation of CAB/RPV LA. Notably, in all patients post-withdrawal data were available, and cessation of CAB/RPV LA led to complete resolution of neuropsychiatric symptoms, irrespective of whether they were rechallenged with an oral INSTI or switched to a non-INSTI backbone. This observation reinforces a causal relationship between LA CAB/RPV exposure and the observed CNS events, while also suggesting that the adverse effects are reversible once plasma drug levels fall below a symptomatic threshold. From a clinical standpoint, these findings underscore 2 practical considerations: (1) early recognition of neuropsychiatric toxicity is critical, as timely discontinuation can prevent prolonged morbidity despite the long pharmacokinetic tail of injectable agents, and (2) prior oral INSTI intolerance does not uniformly preclude successful re-introduction of the same oral agent after LA therapy, although careful monitoring remains essential. Together, these data strengthen the argument for neuropsychiatric surveillance and a clearly defined exit strategy whenever CAB/RPV LA is prescribed to individuals with a history of CNS adverse events.

Although our results provide important real-world insights, particularly for a population underrepresented in clinical trials, broader conclusions cannot yet be generalized. Further prospective, multicenter studies with larger sample sizes and longer follow-up are needed to confirm these associations, better delineate risk factors, and refine clinical decision-making regarding the use of long-acting injectable therapy in individuals with a history of neuropsychiatric intolerance to oral antiretroviral agents. Incorporating standardized neuropsychiatric assessments and patient-reported outcomes will be essential in future investigations to more accurately capture the scope, severity, and impact of CNS symptoms in this setting.
